# Glances at the Hospitals

**Published:** 1903-09-19

**Authors:** 


### GLANCES AT THE HOSPITALS.

### VICTORIA HOSPITAL, BURNLEY.

HERE the income was £3,553 and the expenditure £3,651. The average cost per bed occupied was £53 4s. The highest number of patients resident at one time was 82. The total number of deaths was 79, but 22 died within 48 hours of admission. The medical and surgical tables show the numbers suffering from the various diseases, and the number of recoveries or deaths; and this arrangement is also carried out in the table of operations. There were 3 cases of colotomy, of whom 2 recovered and 1 died. For the radical cure of hernia, 23 operations, all being successful. For strangulated hernia, 13, of which 9 recovered and 4 died, the deaths being caused by perforation, exhaustion, and peritonitis. Of gynæcological operations there were 74, and 71 of these were successful. The total number of operations was 759, and there were 23 deaths. The anæsthetic table shows that chloroform was given 715 times, chloroform and A.C.E. mixture 21, A.C.E. 4, and ether twice. In all cases the open method of administration was followed. There was no death, but bronchitis followed on two occasions.

### SHEFFIELD ROYAL HOSPITAL.

THE total income was £7,644 and the total expenditure was £8,998, leaving a deficiency of £1,354 which, as is usual in such cases, was met by drawing on the capital. Legacies, amount of £2,181, were received, and were placed to the capital account. Additions are being made to the hospital and these will cost £20,000, of which sum about one-half has been received. The hospital contained 111 patients at the beginning of the year, and this number with the year's admissions made 1,766 under treatment. The average number of beds occupied daily was 121; and of the total number under treatment, 1,504 were discharged, 128 died, and 134 remained in the wards. The medical and surgical tables are drawn up so as to show the number of patients treated for the various diseases, the number discharged, the number dying, and the number remaining under treatment at the date of the report. When these details are given regarding the ordinary medical and surgical cases, it is rather odd that the table of operations shows no results, and it would have been interesting and instructive to learn the proportion of recoveries in the 18 cases subjected to the operation for appendicitis, in the 7 cases of lupus, the 4 cases of ovariotomy, and so on. Altogether between major and minor operations there were 770. No anæsthetic table is given.

### RADCLIFFE INFIRMARY, OXFORD.

The ordinary receipts amounted to £7,251, and the ordinary expenditure to £8,440. This shows a considerable deficit of income, and it has been going on for years; the adverse balance being met by transferring money from the establishment fund to the general revenue account, but this expedient cannot go on indefinitely. The Governors very plainly point out that unless an additional £500 a year be forthcoming the invested capital will be gradually eaten up, the hospital will be bankrupt, and the cost of maintaining it thrown on the rates. This is a mournful outlook, and it would seem better to keep the expenditure within the income by closing some of the wards and by practising a rigid economy until such time as the inhabitants of Oxford become alive to their responsibilities. The average cost per head for provisions was £14, and the average stay in the hospital was 21 days. In the medical department there were on January 1st 42 patients in residence, and 528 were admitted since that date. Of the total 155 were discharged cured, 168 were discharged relieved, 164 were discharged for other reasons, 43 died, and 40 remained in the wards. In the surgical house 74 were in residence at the beginning of the year, and 1,604 were admitted during it, making a total of 1,678 under treatment. Of these 966 were discharged cured, 192 were discharged relieved, 392 were discharged for various reasons, 75 died, and 53 remained in the wards. We notice that among the medical diseases were 22 cases of pleurisy, 12 of pneumonia, and 22 of phthisis; but that is all we are told about them, as no results of treatment are published. The same may be said of the ordinary surgical cases. It is true there is a table showing the causes of death in all cases, and from this table, if time permitted, some information might be gathered. The operation table is so compiled that we can see at a glance the results of the operations, and it is a pity that similar columns are not put in the ordinary medical and surgical tables. The anæsthetic table is good. Gas and ether were given 786 times, chloroform 143, gas, ether, and chloroform 84, ether and chloroform 9, A.C.E. mixture 33, ether 226, and gas 37.

### CHELMSFORD AND ESSEX INFIRMARY.

The committee regret to report a deficiency of £550. This arises from the facts that the income derived from patients has decreased and so have the donations and collections from churches, while the expenses of repairs, renewals, and provisions have gone up. Under these circumstances the committee appeal for increased subscriptions to meet the ever-increasing cost of carrying on the work of the infirmary. The medical and surgical statistics are very clearly arranged. We only miss an anæsthetic table.

